# Off-the-shelf medication transformed: Custom-dosed metoprolol tartrate tablets via semisolid extrusion additive manufacturing and the perception of this technique in a hospital context

**DOI:** 10.1016/j.ijpx.2024.100277

**Published:** 2024-08-17

**Authors:** Valerie R. Levine, Mattias Paulsson, Maria Strømme, Julian Quodbach, Jonas Lindh

**Affiliations:** a Division of Nanotechnology and Functional Materials, Department of Material Science and Engineering, Uppsala University, Uppsala SE-751 03, Box 35, Sweden; b Department of Women's and Children's Health, Uppsala University, SE-751 05 Uppsala, Box 256, Sweden; c Division of Pharmaceutics, Utrecht Institute for Pharmaceutical Sciences, Utrecht University, Universiteitsweg 99, 3584 CG Utrecht, the Netherlands

**Keywords:** Additive manufacturing, 3D printing, Dose modification, Semisolid Extrusion

## Abstract

Pharmacies are currently unable to stock proper oral dosage forms for pediatric populations. This leads to manipulation of medications or the need to compound specialized medications, which can be a time-consuming process. Using Semisolid Extrusion (SSE) additive manufacturing (AM), specialized medications can be produced in an expedited process from off-the shelf medication in a hospital or outpatient pharmacy setting. In this study, tablets with a desired dose of 5 mg of metoprolol tartrate derived from commercial Seloken™ 50 mg tablets were 3D printed in a hospital setting. Validation testing was done on five batches, highlighting tablets with a high uniformity in mass and dimension, drug content, acceptable microbial assays, and prolonged release during in-vitro analysis. The average drug content found for the tablets was within ±6% of 5 mg for all batches produced. Comparisons were done between the SSE tablets and capsules produced in an external compounding facility, highlighting several positive aspects of SSE-produced tablets beyond simply shortening the production timeline. The SSE tablets printed in this study are characterized by their smaller size, enhanced prolonged release properties, and more uniform drug content across the tested samples. Additionally, interviews with pharmaceutical professionals were conducted to determine the positive aspects of SSE and further improvements to bring this technique as seamlessly as possible into the pharmacy. This study underscores the feasibility of employing SSE in the production of specialized medications within a hospital environment. Furthermore, it highlights the methodological advantages SSE offers over existing production standards, demonstrating its potential to improve pharmaceutical manufacturing in healthcare settings.

## Graphical abstract

**Figure:**
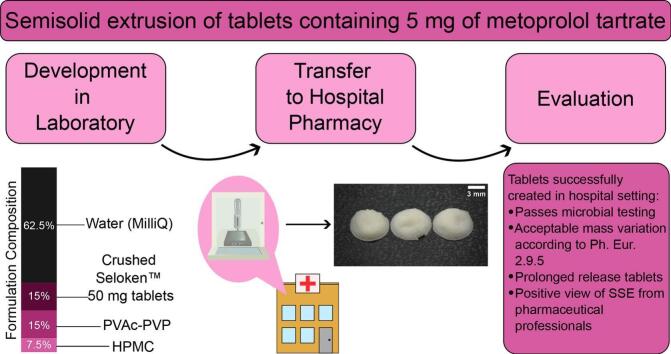

## 1 Introduction

Currently, much-needed medication is not readily available in proper doses for pediatric populations. Pharmacists and caretakers must manipulate conventional oral dosage forms in ways that lack high accuracy (Verrue et al., 2011; Zahn et al., 2020; Richey et al., 2013). If splitting the tablet in a hospital ward is not possible, compounding can occur at a secondary location. In several countries, such as the United Kingdom and Sweden, compounding is often done in a separate pharmaceutical production company or compounding pharmacy, called an external compounding facility (ECF). While EU regulations allow pharmacists to compound in their premises (European Council, 2001), not all countries choose to have compounding within hospital or outpatient pharmacies.

Several countries, such as Germany, allow compounding within hospital and outpatient pharmacies (der Justiz, n.d.). This medication typically takes a day to provide to the patient. While this reduces the wait time from an ECF, creating specialized dosages is still a process that can be improved. Additionally, medications will still need to be manipulated via methods with unknown accuracy (splitting, crushing, etc.) or via capsule filling.

In Sweden, non-aseptic compounding has been centralized to a national unit for the last three decades. This ECF has only one facility producing solid preparations, such as capsules (*Our units - APL*, 2024). Therefore, when a patient needs a specialized oral dosage form this must be ordered through an ordering system with common custom dosages from the ECF's Stockholm facility, no matter where in the country this patient is located. Additionally, Sweden has a convention that medication can only be provided from the hospital pharmacy for approximately three days. This medication is dispensed from a ward as a small quantity to cover a patient's needs until the medication can be dispensed from an outpatient retail pharmacy (*Läkemedelsverkets föreskrifter (HSLF-FS 2019:32) om förordnande och utlämnande av läkemedel och teknisk sprit; | Läkemedelsverket. Läkemedelsverkets föreskrifter (HSLF-FS 2019:32) om förordnande och utlämnande av läkemedel och teknisk sprit*, 2024). This is also true for more countries, e.g., Germany. Therefore, when a patient leaves the hospital, they must reorder this medication from an outpatient pharmacy. If this is specialized medication from an ECF, it must be reordered and remade. On average, orders from an ECF take five days to arrive between order time and arriving to a patient (four days from an ECF and one day from the supplier) (Produktion and Laboratorium, 2009). Adult patients who require common drugs can receive these quickly, since licensed medications are often readily available without the need for specialized compounding. Children, however, are not afforded the same level of comfort.

Often, commercial tablets are manually reformulated to capsules following defined procedures. There is no guarantee, however, that the mixture is homogeneous. While this is true of any method, it is particularly difficult with powders, where particle size, the method of mixing, and possible agglomeration or de-mixing are some of the factors that could impact the mixture homogeneity (Venables and Wells, 2001); Massol-Chaudeur et al., 2002. For capsules developed from commercial tablets, in particular, it has been found that a loss of drug during the compounding step can occur, particularly for oral powder content lower than 300 mg (Helin-Tanninen et al., 2007; Helin et al., 1998). Moreover, the capsule boards employed require thorough cleaning between the processing of different powder formulations to prevent cross-contamination (Haider, 2010; Greene and Ratcliff, 2017).

Using an additive manufacturing technique (AM) called semisolid extrusion (SSE) (sometimes called pressure-assisted microsyringe printing) (El Aita et al., 2019) the workflow of specialized oral dosage form production could improve greatly from the existing setup. In SSE, a semisolid formulation is extruded through a nozzle (Hopson, 2022). The formulation is extruded layer-by-layer in a desired shape dictated by a digital file and slicer software with drying after printing. Many SSE printers are designed for printing biological materials, such as cells. Therefore, they are often designed with sterility and cleanability in mind (Sen et al., 2023; Rahman and Quodbach, 2021). These printers can potentially lack some of the benefits of traditional SSE, such as full shape and size variation, as well as tailoring of the formulation. For in-line control, printing can be done on a mass balance, giving a resultant mass for each tablet. Commercial “inks” that comprise everything but the API are sold, which results in non-modifiable formulations, where features such as viscosity and materials are locked in. For formulations with variation in drug-release time, this could be an undesirable feature.

SSE allows for modifications in the shape, size, formulation, and release of the oral dosage form being printed. The drug release can be controlled by the materials used in the formulation (El Aita et al., 2020; Tagami et al., 2019) or the dosage form geometry (Vlachou et al., 2023; Cui et al., 2021), where different surface area can change the release profile of the dosage form. The shape selectivity possible with SSE also allows for smaller medications compared to the confined dimensions of hard capsules, the current standard for specially compounded medications. Since pediatric patients are able to swallow smaller oral dosage forms more easily (Mistry and Batchelor, 2017), SSE could allow for higher acceptability in this patient group (Saito et al., 2019; Ranmal et al., 2016). Chewable dosage forms (Zhu et al., 2022; Gkaragkounis and Fatouros, 2023; Rodríguez-Pombo et al., 2024) and mini-tablets (Suárez-González et al., 2021) are possible to make using SSE, showing the width of range possible for the texture and size modification of this technology. In addition to this attribute, many SSE printers have multiple print heads, making it possible to print polypills (Khaled et al., 2015; Haring et al., 2018).

SSE is advantageous for use in a hospital or outpatient pharmacy setting for many reasons. First, SSE printers are typically small, fitting easily on an existing benchtop space. A vacuum oven for drying can fit below the benchtop. Additionally, SSE does not require heating of the formulation, a requirement for both Fused Deposition Modelling (FDM) and Selective Laser Sintering (SLS) (Gibson et al., 2015). This lack of heating is beneficial as it enables the use of heat sensitive APIs (Goyanes et al., 2015; Kollamaram et al., 2018).

SSE is also able to utilize off-the-shelf medications, making the idea of using this technology within a hospital or outpatient pharmacy very attractive. A well-stocked pharmacy has common, commercial medications that can be crushed and added to an SSE formulation within the same location. This technology has the potential to revolutionize the creation of modified dosage forms for patient groups in need by disrupting the current workflow. In order for this technology to rise to its potential, it is vital to study SSE tablets with modified dosages made from off-the-shelf medication in a setting where modified dosages are most needed, which is the goal of the present work.

This study aims to investigate the viability of SSE for tablet production in a hospital setting. The entirety of the printing process, from formulation preparation to printing and drying, was completed in a hospital environment for a commonly-used pediatric API, metoprolol tartrate. Multiple batches were manufactured to determine the consistency of the resultant tablets. The entire process was also carried out by a licensed hospital pharmacist to show that the creation of the formulation and printing is not dependent on one individual. Characterization was then performed on the resultant tablets, investigating the mass and size uniformity of the printed tablets, as well as the solid-state characteristics, drug content, in-vitro drug release, and disintegration properties. Microbial analysis was also performed on the tablets printed in the hospital. Results from the SSE-produced tablets were then compared to capsules produced by an ECF, which were ordered to contain the same amount of metoprolol tartrate. In addition, the printing process was demonstrated to hospital personnel and their opinions on the technique and its possible implementation were recorded and evaluated.

## 2 Materials and methods

### 2.1 Materials

Metoprolol tartrate, in the form of Seloken™ 50 mg tablets (AstraZeneca AB, Södertälje, Sweden), was used as the model API for this study. PVAc-PVP (Polyvinyl acetate/polyvinylpyrrolidone copolymer, known commercially as Kollidon SR®), used as the main matrix agent, was kindly provided by BASF (Ludwigshafen, Germany). HPMC (Hydroxypropyl methylcellulose/Hypromellose, commercially Benecel™ K100 LV PH PRM), which acted as the release modifying excipient, was kindly provided by Ashland (Düsseldorf, Germany). Metoprolol tartrate (Merck Life Science AB, Solna, Sweden) and α-lactose monohydrate (Sigma-Aldrich) were obtained for drug content studies. Capsules with 5 mg of metoprolol tartrate were purchased from Apotek Produktion & Laboratorier (APL) (Stockholm, Sweden) for comparison purposes.

### 2.2 Methods

#### 2.2.1 Formulation preparation

The SSE formulation is found in Table 1. This SSE formulation was determined to be suitable via trials with different percentages of each material. The criteria for determining whether a formulation performed well included mixability to a homogeneous state, ability to load into a syringe for printing, extrudability through the selected nozzle, and printability of the formulation. This printability was indicated by the resultant resolution of the print, which was determined via visual assessment. One common issue observed with less successful formulations was dragging of the material already extruded on the printing surface as the printhead moved or constant streaming of the material from the nozzle without pressure applied.

**Table 1:** SSE formulation composition.

Material	Amount of total batch (%)	Amount of total batch (mg)
Crushed Seloken™ 50 mg tablet	15	900
PVAc-PVP	15	900
HPMC	7.5	450
Water (MilliQ)	62.5	3750
Total	100	6000

The formulation was prepared based on the protocol reported by El Aita, et al. (El Aita et al., 2020) First, the metoprolol tartrate tablets were crushed with a mortar and pestle for three minutes and the respective amount was added to deionized water. Due to the non-water-soluble excipients microcrystalline cellulose, sodium starch glycolate, and magnesium stearate in the Seloken™ 50 mg tablets, a clear solution was not obtained. After the addition of the crushed tablet to the water and thorough mixing with a mortar and pestle for an additional three minutes, the PVAc-PVP was added to the solution at room temperature with further mixing via mortar and pestle. This mixing step lasted five minutes. Next, the HPMC was added to the solution and mixed together with a mortar and pestle for five minutes. The precursor could then be loaded into 3 mL printing syringes and stored at room temperature until printing was performed. The formulations were prepared in the morning and the printing took place in the afternoon on the same day. Since HPMC is a hydrophilic polymer, it forms a gel in water (Schmidt, 2019). Therefore, the time between formulation preparation and printing was kept as similar as possible to ensure similar rheological properties and proper hydration between the different batches.

#### 2.2.2 Tablet design

Since specific dosages of the API were desired, the tablet dimensions were designed around the intended dosage. Besides the API, Seloken™ 50 mg tablets contain various excipients (lactose monohydrate, microcrystalline cellulose, sodium starch glycolate, anhydrous non-colloidal silicon dioxide, povidone, magnesium stearate). Therefore, determining tablet dimensions for a specific drug content involved further analysis.

**Fig. 1:**
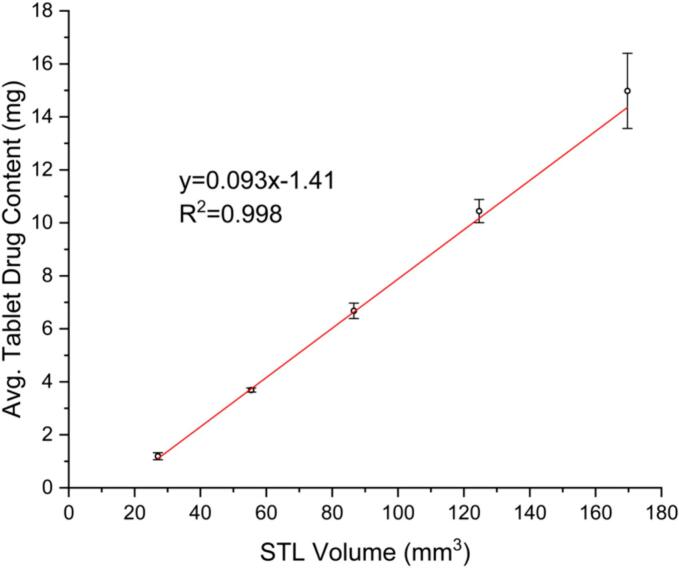
Linear correlation for drug content of tablets with varying STL dimensions for the formulation prepared in this study (x̅ ± s, *n* ≥ 5).

The relationship between tablet dimensions and drug content was investigated to determine the final dosage form dimensions. The tablets are printed from the same formulation. Therefore, tablets printed with different STL volumes should lead to tablets with linearly correlating drug contents. The resulting plot is shown in Fig. 1 (R^2^ of 0.998). This method can be further applied to any formulation and allows for a precise determination of the STL volume before the printing starts. The only limitation is that the stereolithography (STL) file created must be divisible by the nozzle diameter of 0.41 mm (22 gauge). The models for the tablets were designed in Fusion 360 (Student Edition, Autodesk, USA). A cylinder was chosen as the base shape, due to the swallowability of this shape. It is, additionally, a familiar shape from conventional oral dosage forms, which is advantageous for adoption of additive manufactured oral dosage forms.

Tablets with an intended dose of 5 mg were chosen as the model tablet for this study. Using the linear correlation created to determine the appropriate STL volume (Fig. 1**)**, the tablet size fitting this volume was chosen. The dimensions of the tablet selected for this study are d = 7.38 mm, h = 1.64 mm.

#### 2.2.3 Additive manufacturing of tablets

The model was uploaded as an STL file to the BioX™ 3D printer (Cellink, Gothenburg, Sweden), which was used for the semisolid extrusion of the prepared gel formulation. The slicer software (Heart OSTM) integrated into the BioX™ 3D printer was utilized for setting printing parameters. Printing was performed at room temperature. The printhead used was a piston-type syringe pump printhead. The parameters used for printing are found in Table 2.

**Table 2:** Print parameters.

Parameter	Selected setting
Nozzle diameter	0.41 mm (22 gauge)
Extrusion Rate	1 μL/s
Retraction Rate	100 μL/s
Print speed	5 mm/s
Preflow delay	0 ms
Postflow delay	0 ms
Infill pattern	Grid pattern, 99% infill density
Layer thickness	0.41 mm
Number of Layers	4

These print parameters were chosen via trial and error for the selected formulation. Post-printing, drying in a vacuum oven overnight at 40 °C and 400 mbar was performed to ensure that the tablets were fully dry. Batch sizes were 27–30 tablets.

#### 2.2.4 Characterization of the tablets

##### 2.2.4.1 Size and mass measurements

The dimensions and weights of the printed tablets were measured post drying using a digital caliper (digital sliding caliper 150 mm stainless steel, Distrelec Group AG, Nänikon, Switzerland) and weighed using an analytical balance (Mettler Toledo XS 64 Analytical Balance, Schwerzenbach, Switzerland). All tablets were weighed and measured, with the average values reported (x̅ ± s, *n* ≥ 27).

##### 2.2.4.2 Solid state analysis

Differential scanning calorimetry (DSC) thermograms were obtained with a Mettler Toledo DSC 3+ (Schwerzenbach, Switzerland) and a heating and cooling rate of 10 °C min^−1^ and nitrogen as a purge gas. Heating-cooling measurements were carried out from 25 to 125 °C and from 125 to 25 °C in the first cycle, and from 25 to 125 °C in the second cycle. A pinhole method with standard aluminum crucibles (40 μL, DSC Consumables, USA) was used.

Scanning electron microscopy (SEM) was carried out using a Zeiss LEO 1550 (Germany) with Oxford AZtec EDS. Sputter coating of the samples was required, due to their nonconductive nature, using a Polaron SC7640 (East Sussex, UK), where the samples were coated with Pd/Au. An electron high tension voltage (EHT) of 1.00 kV and a secondary electron detector were used.

##### 2.2.4.3 Disintegration testing

Disintegration testing was performed on a PTZ Auto EZ fully automated tablet disintegration testing apparatus from Pharmatest (Hainberg, Germany). Testing was done in accordance with Ph. Eur. 2.9.1 Disintegration of Tablets and Capsules Test A. Following this protocol, six tablets were tested and the average disintegration time is reported (x̅ ± s). 450 mL of 37 °C MilliQ water was used as the disintegration medium.

##### 2.2.4.4 Drug content

Drug content analysis was carried out using UV–Vis (1800, Shimadzu Corporation, Kyoto, Japan) at 274 nm. A calibration curve, based on a 50% (*w*/*v*) metoprolol tartrate stock solution, was created each day of quantification. Additive manufactured tablets from each batch (*n* = 3) were weighed and dissolved in water (50 mL of deionized water for each individual tablet), and filtered through a 0.45 μm PTFE filter. The resultant concentration was determined using a standard curve with an R^2^ > 0.995. The drug quantity could then be determined. Initial quantification was performed in triplicate to ensure consistency between the calibration measurements.

For the drug content of the comparison capsules, the drug content of 20 capsules was measured using the same equipment and procedure as above. In this case, however, the capsules had their gelatin capsule removed and the powder was weighed and dissolved in water (50 mL of deionized water for each individual capsule).

##### 2.2.4.5 In vitro drug release

Drug release studies were performed on the SSE-produced samples, as well as the ECF capsules for comparison. The tests followed the USP II method with a Sotax AT7 (Sotax AG, Aesch, Switzerland) dissolution tester. Each vessel was filled with 900 mL of phosphate buffer, pH 6.8 of at 37 °C, stirred at 100 rpm. 5 mL sample aliquots were removed and filtered with 0.45 μm PTFE filters at predetermined time intervals (5, 10, 15, 20, 30, 45, 60, 90, 120, 180, and 210 min for SSE tablets and 2, 3, 5, 10, 15, and 30 min for ECF capsules). The medium was not replenished between sampling, and this was accounted for when calculating the drug content of the sample. All experiments were performed in triplicate. The percentage and mass of the drug released was calculated via measuring the sample aliquots with UV–Vis spectroscopy. The drug release profiles were investigated for fit with several kinetic profiles using KinetDS 3.0 (Krakow, Poland) software (Mendyk et al., 2012).

##### 2.2.4.6 Microbial assays

Microbial testing was performed using Ph. Eur. 2.6.12 Microbial Examination of Non-sterile Products: Microbial Enumeration Tests as a guideline. Tablets with a total mass of approximately 1 g were added to 10 mL of buffered sodium chloride-peptone solution and dissolved, as the tablets were too hard to crush with readily available methods. Due to the high turbidity of the 1/10 dilution, samples were further diluted 1:1 to 0.05 g/mL. A quantity of 1 mL sample solution was then added to two sterile petri dishes each. For a negative control, 1 mL of buffered sodium chloride-peptone solution was added to a sterile petri dish. Molten tryptic soy agar (TSA) (20–30 mL) were added to each plate and mixed. The plates were then left to set until reaching room temperature. Then, incubation of the plates occurred at 30–35 °C for 3–5 days. The total aerobic microbial count (TAMC) was then taken for each plate and multiplied by 20 to calculate the number of cfu/g.

#### 2.2.5 Comparison to current compounding methods

Comparisons were made with capsules produced by Apotek Produktion & Laboratorier (APL) (Stockholm, SE). An order of 5 mg metoprolol tartrate capsules was made to compare with the tablets produced via SSE that were designed to have 5 mg of metoprolol tartrate. Comparison was done with regard to time of preparation, mass analysis, drug content, and in-vitro drug release.

#### 2.2.6 Batch of printed tablets prepared by pharmacist

One batch of tablets was produced, from start to finish, by a licensed hospital pharmacist, with supervision from the first author of the study. The formulation and printing was all be performed by a pharmacist and the mass, drug content, and dimensions of the resultant tablets was compared to the other batches produced in the study.

#### 2.2.7 Interviews with experts

##### 2.2.7.1 Interview setting and interview recruitment

Five individuals were interviewed for this study at Uppsala University Hospital. Their backgrounds range from two-year and three-year (BSc) university pharmacy degrees to five-year (MSc) and PhD pharmacy degrees and technicians. All participants work in the hospital and have a role in medication handling, making them an appropriate choice for interviewing.

The main interviewer (first author) had never met any of the participants being interviewed prior to the interview day, with the recruitment handled by the co-interviewer (second author), who does know those being interviewed. Interviews were carried out in the library of the hospital, which was a quiet space nearby the SSE printer. The main interviewer introduced SSE and the motivation for the study. Then, the interviewees were shown the SSE printer and had the opportunity to try printing. After, the interviews were conducted.

##### 2.2.7.2 Interview content

Hospital professionals were interviewed for their perspective on SSE of tablets in the hospital setting. The interviews were recorded and transcribed using Microsoft Teams so that the interviewees would remain anonymous for their privacy protection. The base questions of the interviews are found in Table 3.

**Table 3:** Guide for interviews used in this study.

Topic	Question
SSE Production Steps and Workflow	What are your thoughts on the production process that utilizes SSE print technology?
SSE Production Steps and Workflow	Would it be beneficial to have the formulation gel already made instead of creating it yourself?
Potential Benefits of SSE	What do you think are the potential benefits of SSE (for pharmacists, patients, and healthcare professionals)?
Potential Risks of SSE	What do you think are the potential risks of SSE (for pharmacists, patients, and healthcare professionals)?
Usability of Equipment	How did you do when using the printer and what comments do you have on the overall usability?
Usability of Equipment	Is there anything that should be developed further?
Patient Safety	What do you think of the quality of the SSE-manufactured drug/tablet?
Patient Safety	Are there any aspects that should be considered/further developed from a patient safety perspective?
Occupational Safety	How do you think this manufacturing method would impact occupational safety and ergonomics for you and your colleagues?
Future	What new skills would you need if SSE were introduced as a production method for oral medicines?
Future	Should this new technology be adopted?

##### 2.2.7.3 Interview data analysis

Analysis was done post-interview from transcripts and audio recording of the interview sessions. Information was gathered with conclusions being made on the overall interview answers (Braun and Clarke, 2022).

## 3 Results and discussion

### 3.1 Size and mass measurements

**Fig. 2:**
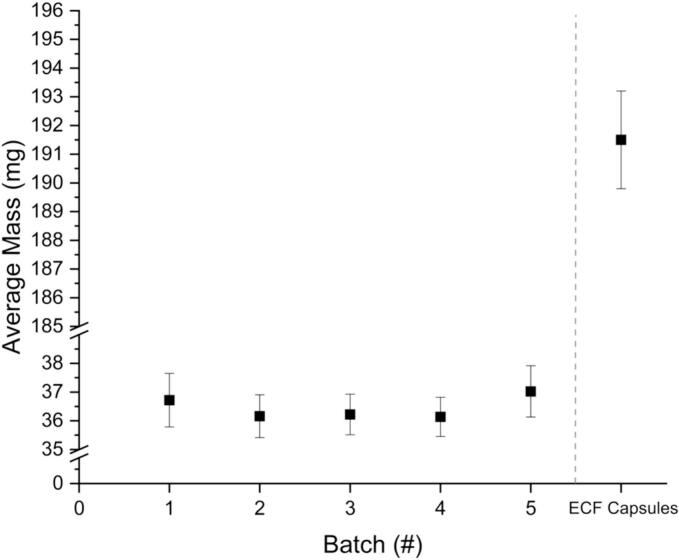
Mass results of five batches of printed tablets (x̅ ± s, *n* ≥ 27) as well as the ECF prepared capsules (x̅ ± s, *n* = 100).

The size and mass of the tablets were determined after the printing process was complete. The results of the mass measurements of the tablets can be found in Fig. 2. Batch five was printed by a licensed pharmacist, the second author of the publication, while the other four batches were printed by the first author. While batch five has a slightly higher average mass than the other batches, it is within the standard deviation of the other batches. Additionally, using statistical t-testing and f-testing, there was no significant difference found between the batches. This indicates no producer dependency. This is important for validation of SSE in a hospital setting, as the process should be consistent, regardless of the individual performing it. The standard deviation for these batches, additionally, was below 1 mg in all cases, indicating a consistency in printing. This signifies that the printer used, as well as the print method and formulation, can yield consistent result with regard to tablet mass.

Ph. Eur. guideline 2.9.5 Uniformity of Mass of Single-Dose Preparations dictates that tablets weighing 80 mg or less cannot have more than two tablets with deviations >10% for the mass. If any one tablet exceeds 20% mass deviation, then the batch additionally fails. No tablets exceeded this 10% mass deviation in the batches produced for this study, indicating that these tablets meet the Ph. Eur. guidelines set out for traditional tablets.

The mass of the capsules is high relative to that of the SSE tablets, with the average mass over five times greater. The relative standard deviation achieved in the capsules is low with 0.9%, caused by the high absolute weight. In comparison, the SSE tablets have increased relative standard deviation ranging from 1.9% to 2.5% for the five batches due to a more than five times lower weight. When analyzing the dimensions of the capsules from the ECF, they are relatively large for a pediatric population, with a capsule size of 4 (lock length of 14.3 ± 0.76 mm). This is significantly larger than the tablets produced via SSE, and with a much lower overall API loading percentage.

**Fig. 3:**
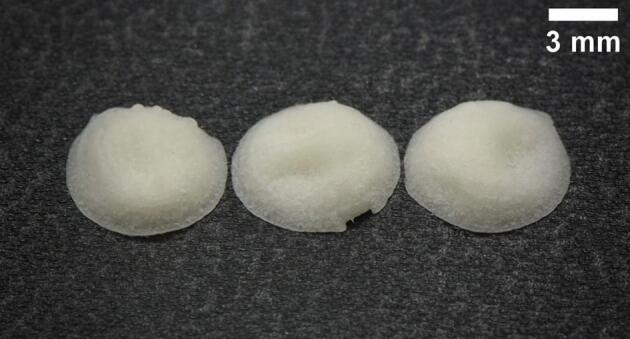
Image of tablets post-drying.

The dimensions of the printed tablets are close to the STL design. The resultant average diameter for all of the batches was 7.2 ± 0.2 mm and the height 1.7 ± 0.2 mm While the resultant dimensions do not exactly match the digital model dimensions of 7.38 mm in diameter and 1.64 mm in height, they are close. The general trend observed is that the tablets have a smaller diameter and more height in actual printed results than in the digital model. This is likely due to the high water content of the tablets, which did not hold the shape of a cylinder post-printing, indicating a too low viscosity. A grid pattern was printed, giving a characteristic additive manufactured look to the tablets, seen in various types of extrusion printing. These lines are visible immediately after printing. Over the course of time, the material coalesces forming a structure where the printing pattern can no longer be discerned. The tablets lack a uniform, cylindrical shape. Dried tablets are shown in Fig. 3.

### 3.2 Solid state analysis

#### 3.2.1 DSC results

**Fig. 4:**
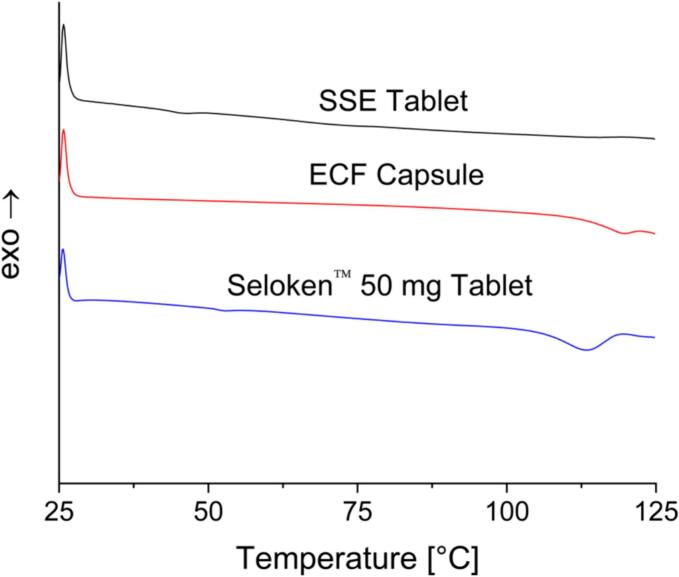
DSC results for SSE tablets, ECF capsules, and Seloken™ 50 mg tablets.

DSC analysis was performed on the Seloken™ 50 mg tablets, the content of an ECF capsule, and the SSE-produced tablets (Fig. 4). The Seloken™ 50 mg tablet shows a small T_g_ at around 50 °C and a T_m_ indicative of metoprolol tartrate at around 113 °C. The T_g_ observed in this thermogram is likely the result of the excipient macrogol (Djekic et al., 2017). The ECF capsule also shows a T_m_ for the metoprolol tartrate at around 120 °C. As now a melting peak is observed for lactose, it is likely that the lactose monohydrate powder in the ECF capsule is amorphous. Looking at the SSE tablets, the T_g_ of the polymer used for the SSE tablets is approximately 35 °C (Bühler, 1992), which is observed to a small degree in the thermograms. The SSE tablets did not show a T_m_ for the metoprolol tartrate, indicating a clear difference. Even though pure metoprolol tartrate is often reported as crystalline (Luch, 1983), no crystallinity peaks were observed. This is consistent among all three thermograms.

#### 3.2.2 SEM results

Analysis via SEM was done on randomly selected tablets made with the selected formulation. The surface of the tablets had a rough texture, seen in **Fig. S1**. It is possible that this texture is a result of the high water content in the formulation, which is eliminated during the drying step. A similar looking surface was observed in other studies using SSE (El Aita et al., 2019; Krishnan et al., 2024; Cheng et al., 2021). It is also possible that the topology is a result of the non-dissolved particles that became “coated” with the dissolved polymer during drying. There is no indication of a porous network in the tablets investigated, which could factor into how the API releases from the tablet.

### 3.3 Drug content

**Fig. 5:**
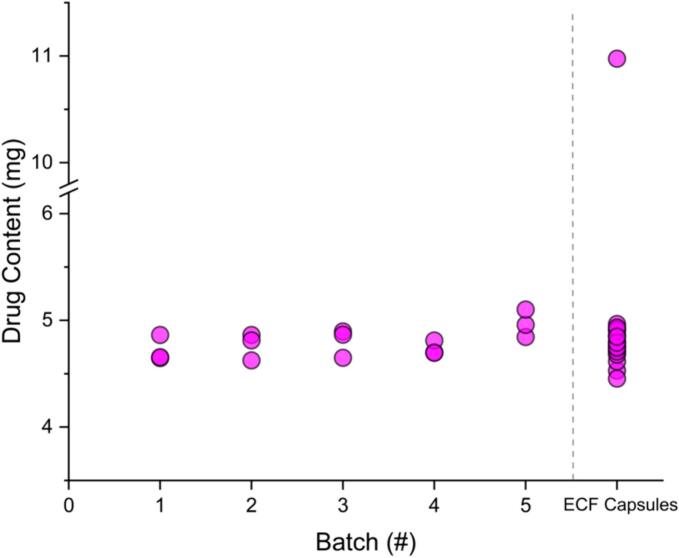
Drug content for tablets tested from the five batches made in the study and the ECF capsules.

The drug content of tablets from the five batches and the ECF capsules can be seen in Fig. 5. While the average drug content is slightly higher in batch five, this batch also had a higher average mass. The amount of drug does not vary much between batches, however, and the average drug content is always within a ± 6% deviation of 5 mg, with an average drug content over the five batches of 4.8 ± 0.1 mg. The tablets randomly selected for drug content testing from batch five were approximately 1 mg heavier than the randomly selected tablets from the other batches. While this random selection resulted in particularly high mass tablets from batch five, they were within a 10% mass deviation from the average mass.

Comparing the SSE tablet data to results of 20 ECF capsules that were randomly selected, the drug content is relatively consistent to the intended 5 mg in both the capsules and tablets (Fig. 5). However, one capsule tested had a drug content of 11.0 mg. This is over twice the expected drug content. The average drug content for the 20 capsules was 5.1 ± 1.4 mg. Excluding this capsule, the drug content was 4.8 ± 0.1 mg, which matches the drug content found for the SSE tablets. While only one of the 20 capsules investigated had a drug content that did not fit the desired amount, it is an alarmingly high drug content. Powder clumps were observed in the capsules when removing the powder for analysis. This clumping could indicate aggregation of drug in some capsules (Venables and Wells, 2001). The mass of the capsule containing 11.0 mg did not reflect this increase in drug content, as this capsule had a mass of 195.6 mg compared to the average capsule weight of 191.5 ± 1.7 mg. If this extra mass was purely drug, however, it would account for a large amount of the additional drug content observed. Comparing this to the relatively consistent results found using SSE, no tablets of this drug content deviation were observed, with all drug contents close to the 5 mg target dose.

### 3.4 In vitro drug release

**Fig. 6:**
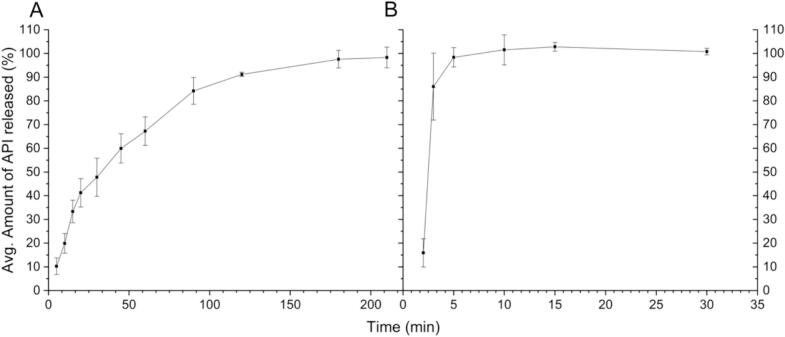
In-vitro drug release, with A) Average Amount of API Released (%) for SSE Tablets, B) Average amount of API Released (%) for ECF Capsules; x̅ ± s, *n* = 3.

Fig. 6 shows the dissolution data of the tablets. The API release from the tablets was approximately 50% within 30 min and was complete within 210 min, displaying prolonged release. Commercial, commonly prescribed tablets with an API of metoprolol tartrate are also prolonged-release tablets (Hainer and Sugg, 2007; *SelokenZOC® - FASS Vårdpersonal*, 2024). The metoprolol tartrate containing tablets commonly prescribed to patients have a prolonged-release with zero-order kinetics with full release in 20 h (*SelokenZOC® - FASS Vårdpersonal*, 2024). While the release found in the SSE tablets is faster and does not follow a zero-order kinetic, it is slower than in the ECF capsules. The drug release for the SSE-produced tablets fit best to logarithmic model, with an R^2^ value of 0.9895, followed by a Higuchi model with an R^2^ value of 0.9320. The Higuchi model is a logical choice for fitting to the data produced for the SSE tablets, as this model was designed for drug release from a matrix system. The ECF capsules showed immediate release characteristics, with a full release of the API within 30 min. For pediatric populations, an immediate release dosage form is often not ideal, as many children struggle with swallowing medications (Diamond and Lavallee, 2010; Jacobsen et al., 2016). Therefore, the API can release prior to the intended time. While some products are used to coat tablets or capsules to make them easier to swallow (Uloza et al., 2010; El Edelbi et al., 2015), this is an additional step that must be taken and another product stocked.

### 3.5 Disintegration testing

Disintegration testing results yielded an average disintegration time of 23.8 ± 6.7 min for the SSE tablets. This is consistent with the in-vitro drug release data. The disintegration did not occur immediately upon exposure to the water during testing, as would be seen for immediate release tablets, but rather as a slow erosion over time. The surface of the tablet allows for significant release, or burst release, even before tablet disintegration can occur to a high degree. This is because the drug particles are directly exposed to the surrounding medium and this is likely the reason there is some drug release observed in the in vitro drug release starting from the first measurement at five minutes.

### 3.6 Microbial assays

Microbial testing of the tablets yielded a total aerobic microbial count (TAMC) of 50 cfu/g. According to Ph. Eur. Table 5.1.4–1, non-aqueous preparations for oral use fall under Ph. Eur. category of 10^3^ for TAMC, meaning that the tablets produced in a hospital setting are acceptable according to microbiological standards. The production of these tablets was done in the existing hospital workflow. No specialized analytical balances, rooms with laminar airflow cabinets, or sterile compounding approaches were employed, with only climate control in the printing room regulating the air. This investigation shows the viability of creating SSE tablets with off-the-shelf medication with regard to the microbiological safety of the product.

### 3.7 Interview with pharmaceutical professionals

The interview information was subdivided by themes for a clearer understanding of the information gathered. The themes include a desire for premade formulations, addition of status indicators, the importance of a labelling system, the wish for an easy-to-use interface, impressions about SSE in a hospital setting, as well as their overall view on the place of SSE technology in hospitals.

#### 3.7.1 Premade formulations

The first theme discovered during the interviews was that the involved personnel prefers to have the formulation material premade. The desired setup by all was to have pre-loaded syringes ready to use, and not to prepare them in the pharmacy or wards. This would, in the view of those interviewed, speed up the process of SSE to an acceptable level and fit the process better into the existing workflow. Creating the formulation was more concerning for those working with cytotoxic drugs where there are more severe occupational health issues.

A possible solution to this is to have the pharmacist in charge of compounding in the central hospital or outpatient pharmacy prepare the formulations or to have ECFs perform this process and provide the formulation. In nations where ECFs currently provide specialized medications, the formulation material could be prepared and bought from the ECF. The speed of patients receiving medication is not hindered by the current system, as formulation intermediates could be kept in stock in a hospital pharmacy. While this process would require further testing of the stability of the formulation, it is an important issue for the acceptability of this method within a hospital framework.

#### 3.7.2 Addition of status indicators

Another aspect discussed was the importance of knowing as efficiently as possible when the status of the printer changes, whether this was when printing was complete, the syringe was empty, a printing error had occurred, etc. Some of those interviewed suggested a noise as an indicator, but others disagreed and asked for a light system, as it is less intrusive. One such comment stated, “…you can have a red light when it's working. [You can have a] green light or something to show that [the status has changed]…for efficiency. [You can then] come and get [the tablet].”

#### 3.7.3 Importance of a labelling system

When analyzing the potential method, those interviewed stated their desire for an added layer of security to avoid mixing up tablets both during choosing of the tablets for a particular patient and printing. They suggested a labelling system be added, whether it be a physical label added to a petri dish full of one type and dose of drug, or a digital system.

One interviewee brought up that, “…if you could connect the 3D printer to a patient…and the data system …where the doctors prescribe things, that would be nice. They could choose and you could get on the screen the patient. You choose the patient and you choose whatever it is, one milligram [dose, for example], and you already have a set program in the 3D printer that is one milligram and it's connected.”

Based on what was said by the interviewees, to avoid errors both in the medication handling process including the printing process and administration, a closed-loop medication management (CLMM) system with, e.g., proper labelling is recommended (Shermock et al., 2023). This process would involve a more robust labelling system, involving automation and intelligent systems to reduce the risk of medication errors (Shermock et al., 2023; Austin et al., 2018; Alotaibi and Federico, 2017). A process in which a patient has a barcode or RFID with their prescription information at step 1 would allow the pharmacist to see which medications need specialized dosages and are eligible for SSE printing. They may then decide the number of tablets printed for the patient and input this information for printing, which will then be printed on a labeled petri dish to avoid medication mix-ups. This system would be more resilient than the compounding methods in place today.

#### 3.7.4 Easy-to-use interface

Additionally, the interviewees stressed that they require training to use the printer in order to feel comfortable operating the technology, as well as an easy-to-use interface. One interviewee elaborated, “for every new equipment in a pharmacy or manufacturing pharmacy, you need to be trained to understand the equipment.” The interviewee then went on to say, “…that's just ordinary procedures in the pharmacy to work with those things…the challenge will be if it's too complicated. It must be easy to use…” The adoption of SSE in a hospital setting must be user-friendly, with a low-complexity interface. This is a crucial step, as the technology will fail adoption if it is too complicated to use without a high level of training or background in additive manufacturing. During the interviews, a comparison was made several times that a user-interface similar to a coffee machine, listing what is desired that can be clicked with low effort, would be ideal. Predetermined print dimensions for the most common APIs could be loaded into a printer in an idealized scenario.

#### 3.7.5 Impressions about SSE in a hospital setting

Without anything to add or change on the printing process, SSE would help reduce waste, according to those interviewed. Protocol dictates that when a tablet is split or crushed, the remaining medication must be discarded. When ordering from an ECF, minimum order quantities must be considered. For example, the minimum order quantity in the Swedish system is frequently 100 dosage units. If the prescription is less than the minimum order quantity, the remaining dosage units are frequently disposed of after the shelf-life of the compounded formulations as the specific dose strength is not prescribed again. This leads to excessive medical waste.

In addition to reducing waste, those interviewed all agreed unanimously that SSE makes it easier to produce a specific dose for someone who needs non-standard dosages. It is a very tunable process, with the ability to change the shape and size of the tablet with the press of a button. This is particularly useful, as one interviewee said, “for the changing and making of smaller tablets, too, so it's easier to swallow.” This is particularly relevant for pediatric and dysphagia-suffering patients.

When discussing the ergonomics and occupational safety of the method, all interviewees agreed that there was no negative impact this process could have compared to the current workflow. Several interviewees said these aspects would be improved with SSE. They felt that the cleaning process was easy, with a printer that can be cleaned with 70% ethanol and that runs with a UV-light overtop, as well as with disposable syringes and tips. They felt that this process, compared to a capsule filling machine, is much cleaner. If the formulations are provided in syringes, several interviewees stated that utilizing SSE would be safer than what is in the hospital wards currently.

#### 3.7.6 Overall view on the place of SSE technology in hospitals

All interviewees unanimously said that they see a future for SSE in hospital wards, and would like to see the technology adopted. They had a positive outlook on the technology and, with the insight from these professionals, improvements on the method can be made for a smooth and successful adoption into a hospital.

### 3.8 Comparison to current workflow

**Fig. 7:**
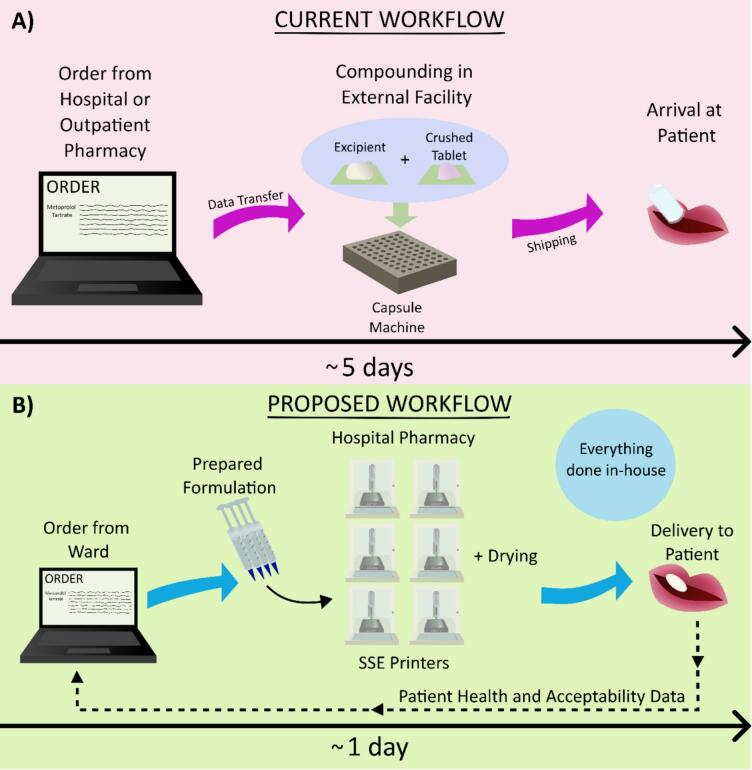
A) Current order process, where an order is placed, then the ECF compounds the capsules, and sends them to the hospital or pharmacy for the patient, with the process taking five days on average, B) Proposed order process, where medication is ordered, printed in the hospital pharmacy, and dispensed out from the centralized printing hub.

Comparing the current workflow with that of the desired workflow incorporating SSE, the process would increase in efficiency. The current workflow when utilizing an ECF takes, on average, five working days (Fig. 7 **A**) (Produktion and Laboratorium, 2009). For this study, from order to delivery, the process was timed to be eight days. While this exact number may vary from country-to-country, it is a major bottleneck for the adoption of personalized medication. As a readily available alternative, some licensed medications can be modified more quickly with manipulation. However, the lower accuracy of this method is also not ideal. The current workflow entails ordering from the hospital or outpatient pharmacy where, in the latter, the data is transferred to the ECF. The medication is then compounded and shipped back to the hospital or pharmacy for a patient's use. Shipping logistics, outside of the direct control of a hospital, pharmacy, or compounding facility, can potentially lead to longer wait times for medications.

While not every country orders specialized medications from an ECF, the creation of specialized dosage forms commonly utilizes capsules, which have been shown in this study to have disadvantages when compared to SSE-produced tablets, or tablet manipulation, which lacks assured accuracy. By centralizing the creation of tablets to SSE in a hospital or outpatient pharmacy, much of the task could be automated in the future. The manpower currently required to compound medications (Silimbani et al., 2019) could be reduced to large-batch formulations being prepared and printed as needed. More rapid production of medications for SSE is even possible, providing an added benefit to this technology (Denis et al., 2024). It is an additional advantage that an SSE printer is relatively simple to clean, with parts that can be easily sterilized or are disposable. This time-saving effort will add up for a compounding pharmacist.

With perspective from the interviews and the successful production of SSE-manufactured tablets in a hospital environment, an idealized workflow incorporating SSE could be devised, with a simplified depiction shown in Fig. 7 **B**. In this model setup, the central hospital or outpatient pharmacy would employ several SSE printers in-house. Syringes filled with formulation for common APIs would either be prepared on a rotation by pharmacists in the pharmacy or be premade by a decentralized manufacturer. The concept of decentralized manufacturing for AM medications is being investigated worldwide (Seoane-Viaño et al., 2023), making the idea of premade formulation plausible. The hospital or outpatient pharmacy would then receive orders for a certain number of tablets with a specific dose. The printing and drying could occur in the hospital or outpatient pharmacy. Tablets would be labeled via a barcode or written label to best keep track of the medications made. The tablets would then be packaged appropriately and sent out from the central hospital pharmacy throughout the network of different hospital wards or be picked up from the outpatient pharmacy. Further validation could be performed on randomly selected tablets. The use of an in-printer mass balance could help determine outliers. The information on average mass and deviation from this value for the tablets could be logged along with the appropriate dimensions of the digital model. This way, real-time validation could be performed, which is lacking in many drug-manipulation methods currently used. Lastly, patient health and acceptability data could be utilized to further optimize the process.

## 4 Conclusion

This study has demonstrated that SSE has the ability to make the leap from a research laboratory into a hospital setting. From formulation preparation to printing and drying, all steps are possible to carry out within an existing hospital framework. Tablets could be produced with a select, desired amount of API. The masses and dimensions of the tablets proved to be uniform both between and within batches. The drug content in random tablets selected were within a close range to the target API dose, with a desirable prolonged-release found from in-vitro testing. Microbial testing proved that this process can be performed successfully in a hospital environment. Interviews with knowledgeable professionals showed what was important to hospital staff regarding compounded medication, as well as possible improvements to the SSE process. These professionals envisioned a future use of SSE in hospitals.

Future studies could further investigate additional SSE formulations made from off-the-shelf medications with select dosages, helping to build a library of possible formulations on commonly needed, specialized dosages. With these formulations, the minimum drying time required could also be investigated to see the full potential for how quickly tablets can be produced. Formulations with improved rheology could be studied, as well, resulting in tablets of more regular shape post-drying. Additionally, suitable printers could be developed incorporating the benefits of a biological printer, such as UV-light and HEPA filters, and the benefits observed in the first pharmaceutical printers, such as a built-in mass balance. With improvements discovered during the interviews conducted in this study, insights into the needs of hospital professionals, such as a user-friendly interface, an SSE printer with a hospital or outpatient pharmacy in mind can be developed.

The quality-of-care benefits SSE tablets could bring to pediatric patients are profound. Patients could receive smaller, more accurate medication from the central hospital pharmacy to the ward or directly from the outpatient pharmacy, potentially without having to order medications from beyond the building. Patients could receive their medication the next day instead of the five day current average when using an ECF. With this technology, children could be closer to receiving the care that most adults can expect, with necessary medication much more quickly within their reach.

## CRediT authorship contribution statement

**Valerie R. Levine:** Writing – review & editing, Writing – original draft, Investigation, Formal analysis, Methodology, Conceptualization. **Mattias Paulsson:** Writing – review & editing, Validation, Methodology, Conceptualization. **Maria Strømme:** Writing – review & editing, Supervision, Resources, Funding acquisition. **Julian Quodbach:** Writing – review & editing, Writing – original draft, Validation, Supervision, Methodology, Formal analysis, Conceptualization. **Jonas Lindh:** Writing – review & editing, Writing – original draft, Validation, Supervision, Resources, Methodology, Funding acquisition, Formal analysis, Conceptualization.

## Declaration of competing interest

The authors declare the following financial interests/personal relationships which may be considered as potential competing interests.

Jonas Lindh reports financial support was provided by Sweden's Innovation Agency. If there are other authors, they declare that they have no known competing financial interests or personal relationships that could have appeared to influence the work reported in this paper.

## Data Availability

Data will be made available on request.

## Appendix A Supplementary data

SEM images of tablets Image 1
