# Supplementary material for: Off-the-shelf medication transformed: Custom-dosed metoprolol tartrate tablets via semisolid extrusion additive manufacturing and the perception of this technique in a hospital context
Source: Int J Pharm X. 2024 Aug 17;8:100277. doi: 10.1016/j.ijpx.2024.100277 (PMC11388020; doi:10.1016/j.ijpx.2024.100277)
Supplement: Supplementary file 1 — SEM images of tablets [file mmc1.docx]

**Off-the-shelf medication transformed: custom-dosed metoprolol tartrate tablets via semisolid extrusion additive manufacturing and the perception of this technique in a hospital context**

**Supplementary Information**

Valerie R. Levine^1^, Mattias Paulsson^2^, Maria Strømme^1^, Julian Quodbach^3^, Jonas Lindh^1^

^1^Division of Nanotechnology and Functional Materials, Department of Material Science and Engineering, Uppsala University

^2^Department of Women’s and Children’s Health, Uppsala University

^3^Division of Pharmaceutics, Utrecht Institute for Pharmaceutical Sciences, Utrecht University


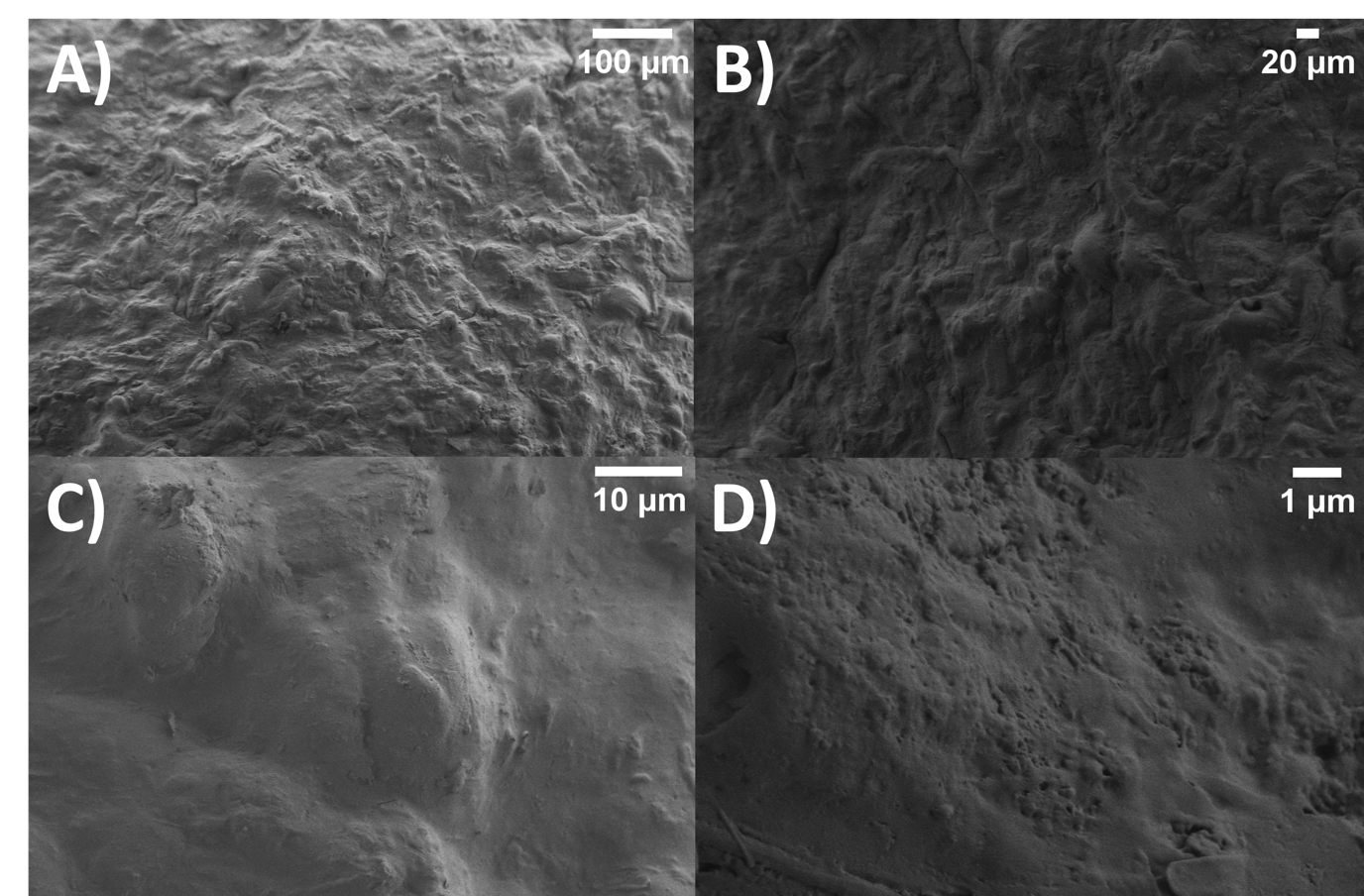


**Figure S1:** SEM images of tablets made with the desired formulation, with A) scale bar of 100 µm, B) scale bar of 20 µm, C) scale bar of 10 µm, D) scale bar of 1 µm
